# Assessing the clinical utility of abdominal computed tomography in sepsis patients with unknown origin: A retrospective cohort study

**DOI:** 10.1097/MD.0000000000038114

**Published:** 2024-05-17

**Authors:** Pei-Hsuan Ho, Yi-Chih Lee, Chip-Jin Ng, Chung-Hsien Chaou, Shou-Yen Chen

**Affiliations:** a Department of Emergency Medicine, Chang Gung Memorial Hospital and Chang Gung University, Linkou, Taoyuan City, Taiwan; b Graduate Institute of Clinical Medical Sciences; Division of Medical Education, College of Medicine, Chang Gung University, Taoyuan City, Taiwan.

**Keywords:** abdominal computed tomography, emergency department, fever, infection source, predictive factors, retrospective study, sepsis

## Abstract

Early identification of the sources of infection in emergency department (ED) patients of sepsis remains challenging. Computed tomography (CT) has the potential to identify sources of infection. This retrospective study aimed to investigate the role of CT in identifying sources of infection in patients with sepsis without obvious infection foci in the ED. A retrospective chart review was conducted on patients with fever and sepsis visiting the ED of Linkou Chang Gung Memorial Hospital between July 1, 2020 and June 30, 2021. Data on patient demographics, vital signs, clinical symptoms, underlying medical conditions, laboratory results, administered interventions, length of hospital stay, and mortality outcomes were collected and analyzed. Of 218 patients included in the study, 139 (63.8%) had positive CT findings. The most common sources of infection detected by CT included liver abscesses, acute pyelonephritis, and cholangitis. Laboratory results showed that patients with positive CT findings had higher white blood cell and absolute neutrophil counts and lower hemoglobin levels. Positive blood culture results were more common in patients with positive CT findings. Additionally, the length of hospital stay was longer in the group with positive CT findings. Multivariate logistic regression analysis revealed that hemoglobin levels and positive blood culture results independently predicted positive CT findings in patients with fever or sepsis without an obvious source of infection. In patients with sepsis with an undetermined infection focus, those presenting with leukocytosis, anemia, and elevated absolute neutrophil counts tended to have positive findings on abdominal CT scans. These patients had high rates of bacteremia and longer lengths of stay. Abdominal CT remains a valuable diagnostic tool for identifying infection sources in carefully selected patients with sepsis of undetermined infection origins.

## 1. Introduction

Sepsis is an aberrant host response to infection, resulting in organ dysfunction. It carries a substantial risk of mortality and is a significant global public health concern, with millions of patients receiving treatment for sepsis annually.^[1–4]^ Epidemiological investigations have revealed a mortality rate ranging from 20% to 50%, with >20% of patients with sepsis requiring mechanical ventilation, thereby imposing considerable financial burdens on healthcare systems.^[5–8]^ Despite advancements in clinical guidelines for sepsis management, the early identification of individuals at an increased risk of sepsis remains a formidable challenge.^[9]^

Fever is a frequently encountered presentation in emergency departments (ED),^[10]^ often attributable to infectious etiologies.^[11]^ Severe infections can precipitate organ dysfunction, which is defined as sepsis.^[2]^ The prompt identification of the source of infection in patients with sepsis is pivotal for timely and precise treatment.^[12]^ This facilitates the administration of antibiotics that target specific pathogens, as opposed to broad-spectrum empirical antibiotics, and enables timely intervention as needed.^[1,2,4]^ However, it is important to acknowledge that not all patients with sepsis have their infectious source accurately identified during their initial presentation to the ED. This poses a significant challenge for emergency physicians.^[13]^

The role of computed tomography (CT) in diagnosing patients with sepsis remains unclear as current guidelines no longer emphasize the use of imaging to identify the source of infection.^[4]^ However, recent sepsis campaigns and other guidelines still acknowledge the valuable role of imaging studies in confirming the potential sources of infection, particularly in cases of suspected intra-abdominal infections.^[1,2,4]^ Furthermore, previously published data have indicated a high positive predictive value of CT in establishing a discharge diagnosis for patients presenting to the ED with suspected sepsis.^[13]^

Nonetheless, limited information is available regarding the indications for performing CT imaging to identify the source of infection in ED patients with suspected sepsis. Additionally, the effectiveness of abdominal CT scans in patients with sepsis who do not exhibit overt gastrointestinal symptoms remains unclear. Previous studies have shown that CT scans are instrumental in confirming or ruling out at least 95% of the alternative diagnoses.^[9]^ However, acknowledging the potential risks associated with CT scans is important, which include allergic reactions to the contrast agents used in contrast-enhanced CT examinations^[14–17]^ and the possibility of worsening kidney function.^[18–20]^ This study aimed to investigate the utility of CT scans in identifying the source of infection in patients with sepsis presenting to the ED with unknown sources of infection. The overarching goal was to pinpoint which patient groups might benefit from abdominal CT and identify the characteristics associated with positive abdominal findings.

## 2. Materials and methods

### 2.1. Study design and settings

We conducted a retrospective cohort study of patients visiting the ED of Linkou Chang Gung Memorial Hospital during the study period. The study site was a tertiary medical center with a 3600-bed capacity and an annual ED visit of 180 000 patients. This study was approved by the Institutional Review Board of the Chang Gung Medical Foundation (IRB No. 202300261B0).

### 2.2. Patient selection & data collection

Data of patients who visited the study site between July 1, 2020, and June 30, 2021, and were eligible according to our inclusion criteria, were extracted from electrical health records. The inclusion criteria were as follows: patients with ED diagnosis of sepsis, based on the International Classification of Diseases code A40.9 (sepsis) or R50.9 (fever), and having undergone abdominal CT scans right after sepsis of unknown origin was diagnosed by emergency physicians. Patients with inaccurate input of the International Classification of Diseases code, with obvious infection focus before CT examination, and with symptoms related to abdominal infection were excluded through a detailed chart review. Besides, patients with fever attributed to various factors, including autoimmune disorders and specific medications, were excluded from the study. Any instances of fever explained by alternative causes, such as heat-related illnesses, endocrine disorders, cancers affecting the immune system or bone marrow, neurological conditions, and post-surgical fever, were systematically excluded through meticulous chart review.

We collected the following data: demographic variables, including sex and age; regularly collected ED presenting information, including vital signs, signs and symptoms, underlying diseases, and laboratory data; and prognostic variables, including provision of interventions (operation or drainage), prescription of inotropic agents, length of admission, admission to the intensive care unit (ICU), and mortality.

Patients with positive CT findings exhibited CT scans revealing acute infectious or new-onset tumor lesions causing inflammatory changes. Conversely, patients with negative CT findings had CT scans showing no acute infectious or relatively stable tumor lesions. Within the subset of patients with positive CT findings, we further categorized them into 2 groups: those who received interventions such as drainage or surgical intervention and those who did not receive interventions.

### 2.3. Statistical analysis

Descriptive results are presented as numbers (percentages) or means (SD), as appropriate. Categorical data between independent groups were compared using the Chi-squared test or Fisher exact test, as appropriate. Continuous data between independent groups were compared using Student *t* test. Multivariate logistic regression analysis was performed to analyze the effects of multiple influential factors on the target outcomes. All data were recorded and processed using Microsoft Excel (Version 17.0, Microsoft, Redmond), and all statistical analyses were performed using SPSS (Version 22, IBM, Armonk. A *P* value of <.05 was considered statistically significant.

## 3. Results

**Figure 1.**
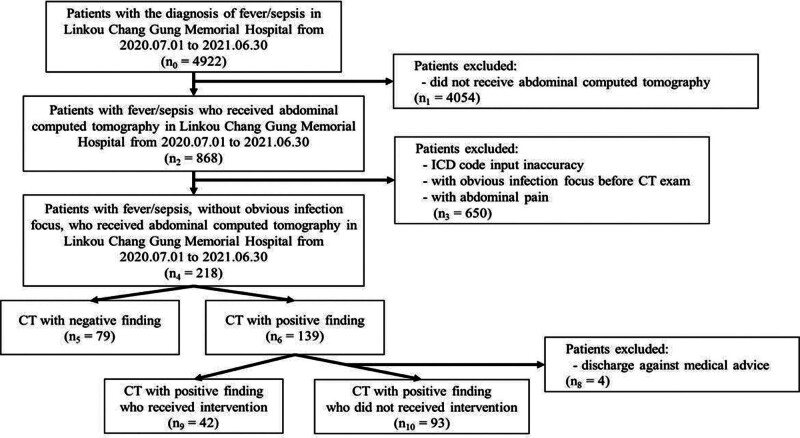
Flow diagram of the study selection process.

**Figure 2.**
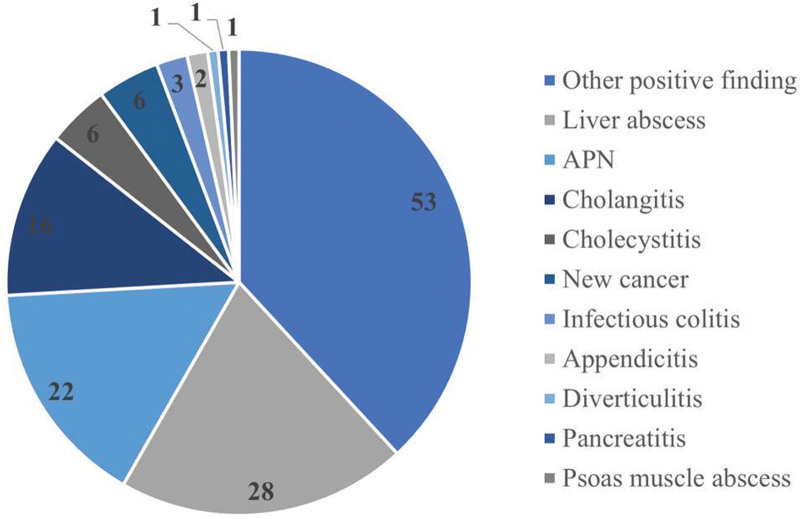
Proportion of infection sources from computed tomography.

A total of 4922 patients visited the ED with a diagnosis of fever or sepsis during the study period, and 218 patients were eligible for inclusion in the study (Fig. 1). Among the included patients, 139 (63.8%) had positive CT findings of infection, whereas 79 (36.2%) did not have an infection focus shown on CT. The most common infection focus found on CT was liver abscess (n = 28, 20.1%), followed by acute pyelonephritis (n = 22, 15.8%) and cholangitis (n = 16, 11.5%) (Fig. 2). Cases presenting challenges in categorization due to uncommon presentations such as abscess formation in rare locations (such as esophageal rupture with mediastinal abscess, retroperitoneal abscess, splenic abscess, etc), less common inflammatory changes like aortitis, mediastinitis, and some post-operative inflammatory changes, as well as instances of cancer progression, were categorized to the part “other positive findings” (n = 53, 38.1%). In the analysis of descriptive results, there was no significant difference among sex proportions, whereas patients with positive CT findings were significantly older than patients with negative CT findings (63.8 ± 16.9 vs 57.8 ± 19.2, *P* = .019). The initial vital signs upon ED visit were similar between the 2 groups. Regarding underlying diseases, there were significantly more patients with hypertension (41.0% vs 25.3%, *P* = .027) with positive CT findings. Regarding laboratory data, white blood cell (WBC) counts (12.33 ± 7.47 vs 10.37 ± 4.58, *P* = .035) as well as absolute neutrophil counts (10266.9 ± 5806.8 vs 8318.22 ± 4386.22, *P* = .010) were significantly higher and hemoglobin (11.9 ± 2.17 vs 12.7 ± 2.22, *P* = .011) was significantly lower in the group with positive CT findings. Positive blood culture results were observed in the group with positive CT findings. Among the group with positive CT findings, the length of stay was significantly longer (15.07 ± 12.56 vs 10.61 ± 11.37, *P* = .010). There were no significant differences in ICU admission rate, inotropic agent use, or mortality rate (Table 1). Univariate and multivariable logistic regression analyses showed that hemoglobin level and positive blood culture were independent predictors of positive CT findings (Table 2).

**Table 1:** Characteristics between computed tomography (CT) with positive and negative findings.

Characteristics		All n = 218	With findings n = 139	Without findings n = 79	*P* value	Normal range, units
Male sex		128 (58.7)	77 (55.4)	51 (64.6)	.201	
Age (yr, mean ± SD)		61.6 (18.0)	63.8 (16.9)	57.8 (19.2)	.019*	
Temperature (ºC)		38.2 (1.1)	38.2 (1.2)	38.2 (1.09)	.987	
Pulse (per min)		105.2 (20.3)	106.9 (20.5)	102.3 (19.9)	.110	
Respiratory rate (per min)		18.5 (2.71)	18.6 (2.91)	18.2 (2.33)	.290	
Oxygen saturation (SpO_2_)		94.9 (2.40)	94.8 (2.40)	95.1 (2.40)	.350	
Systolic blood pressure (mm Hg)		136.6 (28.5)	136.8 (30.2)	136.3 (25.3)	.289	
Diastolic blood pressure (mm Hg)		77.5 (16.2)	77.3 (16.2)	77.7 (16.2)	.852	
Underlying disease						
	Diabetes mellitus	62 (28.4)	39 (28.1)	23 (29.1)	.877	
	Hypertension	77 (35.3)	57 (41.0)	20 (25.3)	.027*	
	Liver cirrhosis	13 (6.0)	8 (5.8)	5 (6.3)	1.000	
	End-stage renal disease	12 (5.5)	7 (5.0)	5 (6.3)	.761	
	Cancer	53 (24.3)	34 (24.5)	19 (24.1)	1.000	
	Autoimmune	3 (1.4)	2 (1.4)	1 (1.3)	1.000	
Laboratory data						
	Sugar	161.7 (88.8)	161.9 (93.9)	161.4 (79.2)	.970	70–140, mg/dL
	Creatinine	1.20 (1.28)	1.23 (1.37)	1.15 (1.20)	.682	Female: 0.44–1.03, mg/dL Male: 0.64–1.27, mg/dL
	C-reactive protein (CRP)	113.5 (84.4)	120.5 (83.8)	101.5 (84.6)	.115	<5, mg/L
	Alanine transaminase (ALT)	87.3 (159.7)	70.7 (96.3)	117.7 (232.8)	.042*	≦36, U/L
	Aspartate aminotransferase (AST)	129.3 (176.6)	107.1 (112.0)	165.7 (248.0)	.223	≦34, U/L
	Total bilirubin	1.72 (1.84)	1.72 (1.93)	1.73 (1.69)	.985	0.1–1.2, mg/dL
	Alkaline phosphatase	158.8 (171.4)	169.2 (179.9)	140.1 (155.0)	.332	19–65 y/o: 28–94, U/L >65 y/o: 40–140, U/L
	Lipase	44.5 (60.7)	51.1 (67.3)	21.0 (10.1)	.252	22–51, U/L
	Lactate	18.0 (13.1)	19.6 (14.5)	15.3 (9.5)	.069	4.5–19.8, mg/dL
	Procalcitonin	30.6 (40.4)	37.0 (42.7)	23.3 (39.6)	.532	Sepsis risk Low: <0.5, ng/mL Medium: 0.5–2, ng/mL High: 2–10, ng/mL Very High: >10, ng/mL
	White blood cell count	11.6 (6.63)	12.3 (7.47)	10.4 (4.58)	.035*	Female: 3.5–11, 1000/μL Male: 3.9–10.6, 1000/μL
	Hemoglobin	12.2 (2.22)	11.9 (2.17)	12.7 (2.22)	.011*	Female: 12–16, g/dL Male: 13.5–17.5, g/dL
	Absolute neutrophil count	9561 (5407)	10267 (5807)	8318 (4386)	.010*	1800–7800, μL
	Positive blood culture	66 (30.3)	52 (37.4)	14 (17.7)	.002*	
Inotropic agent usage		10 (4.6)	8 (5.8)	2 (2.5)	.335	
Length of stay (d)*		13.4 (12.3)	15.1 (12.6)	10.6 (11.4)	.010*	
Intensive care unit admission		21 (9.8)	17 (12.6)	4 (5.1)	.096	
Death		6 (2.8)	4 (3.0)	2 (2.5)	1.000	

* n = 218-4 = 214 (Excluding 4 patients who left against medical advice without available prognosis data).

**Table 2:** Multivariate logistic regression analysis for the prediction of resulting CT findings.

Variable	Univariate			Multivariate		
	Odds ratio	95% CI	*P* value	Odds ratio	95% CI	*P* value
Age	1.019	1.003–1.035	.02*	1.007	0.989–1.025	.469
Hypertension	2.051	1.115–3.772	.021*	1.419	0.688–2.927	.343
White blood cell count	1.064	1.005–1.127	.032*	0.974	0.781–1.214	.812
Hemoglobin	0.845	0.740–0.964	.013*	0.861	0.749–0.990	.036*
Absolute neutrophil count	1.000	1.000–1.000	.011*	1.000	1.000–1.000	.386
Positive blood culture	0.360	0.184–0.706	.003*	0.442	0.210–0.929	.031*

In the subgroup analysis of 139 patients with positive CT findings, 4 (2.9%) patients were discharged against medical advice and were not included in the following statistics. Of the remaining 135 patients, 42 (31.11%) received interventions. Sex proportion, age, or initial ED vital signs did not differ significantly between the 2 groups. There were no significant differences in the underlying diseases between the 2 groups. Regarding laboratory data, patients who received interventions had significantly higher levels of total bilirubin (2.40 ± 2.65 vs 1.47 ± 1.49, *P* = .017) and procalcitonin (57.86 ± 40.50 vs 5.57 ± 9.22, *P* = .050). There were no significant differences in the use of inotropic agents, length of stay, ICU admission rate, or mortality between the 2 groups (Table 3). The results of univariate and multivariate logistic regression analyses showed no significant differences.

**Table 3:** Comparison of characteristics between patients with CT findings who did and did not received interventions.

Characteristics	Intervention n = 42*	No intervention n = 93*	*P* value	Normal range, units
Male Sex	26 (61.9)	48 (51.6)	.350	
Age (yr, mean ± SD)	65.5 (14.7)	62.6 (17.9)	.360	
Temperature (ºC)	38.4 (1.06)	38.1 (1.19)	.142	
Pulse (per min)	107.9 (22.1)	106.2 (19.9)	.656	
Oxygen saturation (SpO_2_)	95.0 (2.37)	94.7 (2.40)	.502	
Systolic blood pressure (mm Hg)	140.0 (30.1)	135.9 (30.4)	.464	
Diastolic blood pressure (mm Hg)	77.0 ± 15.8	78.0 ± 16.4	.748	
Underlying disease				
Diabetes mellitus	14 (33.3)	24 (25.8)	.411	
Hypertension	19 (45.2)	36 (38.7)	.571	
Liver cirrhosis	3 (7.1)	5 (5.4)	.704	
End stage renal disease	1 (2.4)	6 (6.5)	.435	
Cancer	10 (23.8)	23 (24.7)	1.000	
Autoimmune	0 (0.0)	2 (2.2)	1.000	
Laboratory data				
Sugar	163.1 (65.6)	161.6 (106.8)	.933	70–140, mg/dL
Creatinine	1.26 (1.99)	1.20 (1.01)	.818	Female: 0.44–1.03, mg/dL Male: 0.64–1.27, mg/dL
CRP	134.0 (90.0)	115.2 (81.2)	.243	<5, mg/L
ALT	79.0 (74.5)	68.9 (106.5)	.587	≦36, U/L
Total bilirubin	2.40 (2.65)	1.47 (1.49)	.017*	0.1–1.2, mg/dL
Alkaline phosphatase	182.2 (184.9)	168.0 (182.2)	.735	19–65 y/o: 28–94, U/L >65 y/o: 40–140, U/L
Lipase	72.8 (98.6)	34.1 (12.5)	.157	22–51, U/L
Lactate	18.7 (11.4)	18.9 (13.7)	.929	4.5–19.8, mg/dL
Procalcitonin	57.9 (40.5)	5.6 (9.2)	.050*	Sepsis risk Low: <0.5, ng/mL Medium: 0.5–2, ng/mL High: 2–10, ng/mL Very High: >10, ng/mL
White blood cell count	12.7 (5.1)	12.2 (8.4)	.734	Female: 3.5–11, 1000/μL Male: 3.9–10.6, 1000/μL
Hemoglobin	12.1 (1.95)	11.8 (2.29)	.553	Female: 12–16, g/dL Male: 13.5–17.5, g/dL
Absolute neutrophil count (1000/)	10.7 (4.6)	10.0 (6.4)	.508	1.8–7.8, 1000/μL
Positive blood culture	19 (45.2)	31 (33.3)	.248	
Inotropic agent usage	2 (4.8)	6 (6.5)	1.000	
Length of stay (d)	17.3 (12.9)	14.1 (12.3)	.170	
Intensive care unit admission	8 (19.0)	9 (9.7)	.162	
Death	1 (2.4)	3 (3.2)	1.000	

CT = computed tomography.

* n = 139-4 = 135 (Excluding 4 patients who left against medical advice without available intervention data).

## 4. Discussion

Currently, the decision regarding the use of abdominal CT scans in patients with sepsis without a clear source of infection, with the goal of identifying potential intra-abdominal infections, remains a topic of debate. The objective of this study was to delineate specific patient profiles, using their clinical data, that may require an abdominal CT scan. We provide valuable insights that can refine the clinical rationale for employing abdominal CT scans in cases of sepsis in which the source of infection is unclear. In patients experiencing fever or sepsis, swiftly pinpointing the origin of infection facilitates tailored and effective treatments.^[9]^

Previous studies have validated the effectiveness of CT in accurately diagnosing and influencing the treatment course of individuals with fever or sepsis.^[12,21–23]^ A notable prospective study conducted across 4 medical centers underscored the capability of CT in confirming or dismissing at least 95% of alternative diagnoses. This has resulted in revised admission decisions for a significant proportion of patients presenting with symptoms such as abdominal pain, chest pain, dyspnea, or headaches.^[22]^ Additionally, a retrospective analysis focused on ED admissions revealed that CT scans exhibited an 82% positive predictive value in identifying the focal point of discharge diagnosis.^[13]^

Despite blood culture being a relatively slower indicator than other blood tests and lacking the ability to yield immediate results, previous studies have emphasized its pivotal role in the management of patients with fever or sepsis.^[10]^ Positive blood culture results not only signify a more severe infection but also play a crucial role in refining treatment strategies and predicting patient outcomes. This is particularly pertinent in older adults, where blood cultures remain an essential tool for investigating unexplained fevers.^[24]^ Therefore, we recommend that when a patient presenting with fever or sepsis exhibits an inconclusive focus of infection, but subsequently tests positive for blood culture, suggesting a potential intra-abdominal bacterial infection, a follow-up CT scan is warranted. This approach has the potential to significantly affect both treatment decisions and prognosis.^[12]^ Therefore, unless contamination can be confidently ruled out, a positive blood culture typically signifies a genuine underlying infection. When this is coupled with negative results from urine examinations and chest radiography, the likelihood of an intra-abdominal infection increases, explaining the higher frequency of positive findings on CT scans in such cases.

In this study, higher WBC and neutrophil counts were associated with positive CT findings. This observation, which has not been prominently emphasized in previous studies, is significant. Leukocytosis has long been regarded as a crucial indicator for confirming the presence of sepsis,^[25]^ with an elevated neutrophil count considered an even more significant marker for diagnosing sepsis.^[26–28]^ Neutrophils play a pivotal role in the body defense against infections and serve as an initial barrier against microorganisms.^[26]^ A growing body of evidence suggests that restoration of normal neutrophil function can enhance bacterial elimination and improve outcomes in sepsis. This underscores the potential of targeting neutrophils as a viable therapeutic strategy for sepsis.^[27]^ Consequently, patients with fever or sepsis who exhibit positive CT findings typically present more severe intra-abdominal infections, thereby displaying leukocytosis with a left shift in their blood count. Additionally, organ dysfunction resulting from severe sepsis can also contribute to elevated liver enzymes and potentially progress to acute liver failure. Recent studies have also indicated that liver dysfunction can serve as an early indicator of sepsis.^[29]^ In the context of our cohort study, these factors may help explain the observed high prevalence of abnormal liver function tests, which were observed across patients both with positive findings on CT scans and those without any significant findings.

Some patients with positive CT findings may require invasive procedures such as CT-guided drainage or surgical intervention.^[21]^ In our study, among patients receiving interventions, higher total bilirubin levels were noted, corresponding to a higher prevalence of conditions such as liver abscesses or cholangitis that necessitate further intervention. However, elevated bilirubin levels are not inherently linked to positive CT findings, and may not be elevated in patients without hepatic biliary disease. Previous research has shown that patients with amebic liver abscesses are more susceptible to jaundice, with speculations pointing toward hepatic necrosis causing damage to the bile ducts and various vascular structures.^[30]^ Although bilirubin levels may not correlate directly with positive CT findings, they could indicate hepatic biliary disease, for which abdominal CT is the standard diagnostic tool. Therefore, abdominal CT scans for patients with fever, sepsis, and elevated bilirubin levels are still recommended to exclude liver abscesses or other obstructive jaundice conditions that may benefit from invasive interventions, as these procedures can potentially enhance their prognosis.

## 5. Limitation

This study has some limitations. First, owing to its retrospective nature, some data may have been absent or missing from the primary patient management and data collection processes. Furthermore, the study was conducted at a single center, and the relatively limited number of patients could potentially limit the overall generalizability of the findings and hinder our ability to draw definitive conclusions. For more precise results and a comprehensive discussion, conducting a prospective multicenter study in the future should be considered.

## 6. Conclusion

In patients with sepsis with an undetermined infection focus who underwent abdominal CT for survey of origin, those with positive findings tended to be older and presented with leukocytosis, anemia, and elevated absolute neutrophil counts. These patients had high rates of blood culture positivity and longer hospital stays. The most common finding on abdominal CT was liver abscesses, followed by acute pyelonephritis and cholangitis. High levels of total bilirubin and procalcitonin were associated with the need for further interventional procedures in patients with positive abdominal CT findings. Abdominal CT remains a valuable diagnostic tool for identifying infection sources in carefully selected patients with sepsis of undetermined infection origins.

## Acknowledgments

This research was supported by the Chang-Gung Memorial Hospital. We are thankful to our colleagues who provided their expertise, which greatly assisted in the research, although they may not agree with all the interpretations provided in this paper.

## Author contributions

**Conceptualization:** Shou-Yen Chen.

**Data curation:** Pei-Hsuan Ho, Shou-Yen Chen.

**Formal analysis:** Pei-Hsuan Ho, Chung-Hsien Chaou.

**Methodology:** Pei-Hsuan Ho, Chip-Jin Ng.

**Project administration:** Chip-Jin Ng.

**Supervision:** Chung-Hsien Chaou.

**Writing – original draft:** Pei-Hsuan Ho.

**Writing – review & editing:** Yi-Chih Lee, Shou-Yen Chen.
